# Participant recruitment and retention in randomised controlled trials of melanoma surveillance: A scoping review

**DOI:** 10.1016/j.conctc.2025.101461

**Published:** 2025-02-16

**Authors:** Deonna M. Ackermann, Karen Bracken, Jolyn K. Hersch, Monika Janda, Robin M. Turner, Katy J.L. Bell

**Affiliations:** a Sydney School of Public Health, Faculty of Medicine and Health, The University of Sydney, Australia; b Kolling Institute, Faculty of Medicine and Health, The University of Sydney, Australia; c Centre for Health Services Research, The University of Queensland, Brisbane, Australia; d Biostatistics Centre, University of Otago, Dunedin, New Zealand

## Abstract

**Background:**

This scoping review aims to collate and describe data on recruitment, retention, and strategies used to improve these, in randomised controlled trials of melanoma surveillance.

**Methods:**

We searched MEDLINE, EMBASE, CINAHL and CENTRAL databases from inception until October 23, 2023. Two reviewers screened titles and abstracts, and full-texts, and one reviewer extracted data (convenience sample (n = 5) checked by a second). Eligibility criteria included: (i) RCT design, (ii) clinical setting, (iii) participants at increased risk of melanoma, (iv) interventions for early melanoma detection, and (v) early detection outcomes or surrogates such as improved skin self-examination. We calculated summary statistics and undertook qualitative data synthesis.

**Results:**

From 1746 records, 21 trials (reported in 28 papers) were included. Recruitment sources included dermatology clinics, general practice sites, and hospital databases or registries. Trials reported proportions of those screened who were eligible (mean 75 %, range 24–100 %), proportions of those eligible who were randomised (mean 63 %, range 24–95 %), numbers randomised per month (mean 25 participants, range 2–74), and proportion of those randomised who completed outcome measurements (mean 85 %, range 59–100 %) for self-report questionnaires at primary timepoints). Recruitment strategies included targeted participant identification and flexible consent processes. Retention strategies included setting narrow eligibility criteria, reminders, and financial incentives. Reporting on strategies was limited and there were no reports on effectiveness. Few studies reported recruiter facing initiatives or public and patient involvement.

**Discussion:**

More consistent and detailed reporting of recruitment and retention strategies in RCTs is needed, alongside evaluations of their effectiveness.

## 1 Background

Randomised controlled trials (RCTs) play a key role in evidence-based practice but conducting RCTs can be a long, complicated, and resource-intensive enterprise. Research waste is estimated to be 85 %, with preventable weakness in trial design, conduct and analysis identified as a significant cause [1,2]. Causes of research waste in RCTs include inadequate participant recruitment and incomplete outcome ascertainment due to non-retention (withdrawal of consent or loss to follow-up) or non-response to the schedule of follow-up procedures and assessments [3]. Participant recruitment is essential for the delivery of trials, but many trials fail to recruit to time and target, resulting in delays and additional costs [4]. Minimising missing data (from non-retention and non-response) is critical to avoid loss of statistical power and selection bias through differential loss to follow-up between the intervention and control groups [5].

Detailed reporting on these processes, and the trial context, are important for informing future trials, guiding sample size calculations, setting realistic recruitment expectations, determining necessary sites, accounting for loss to follow-up, and understanding resource implications, including staffing and budget. We also need evidence on the effectiveness of strategies to improve trial processes, with methods to improve recruitment and to minimise attrition identified as top priorities in trial methodology [6]. Recent studies and reviews have targeted recruitment-enhancing strategies [7,8] with less focus on retention or response to trial tasks [9,10]. Evidence regarding trial methodology in melanoma surveillance research in particular, remains sparse with most strategies applied in an ad hoc manner.

This scoping review aimed to systematically identify and collate available evidence on how recruitment and retention are measured, and strategies used to improve these trial processes, in RCTs of melanoma surveillance (Population, Concept, and Context is presented in Supplementary Table 1).

## 2 Methods

This scoping review followed the Joanna Briggs Institute methodology for scoping reviews [11] and the protocol was registered in the Open Science Framework [12]. This report adheres to the Preferred Reporting Items for Systematic Reviews and Meta-Analyses extension for Scoping Reviews (PRISMA-ScR) checklist (Supplementary methods) [13].

### 2.1 Search strategy

A limited initial search of MEDLINE (OVID) and SCOPUS was conducted with assistance from an academic librarian. Text words in the titles and abstracts from relevant articles, index terms, and search strategies from related reviews [7,10] were used to develop a full search strategy (Supplementary Fig. 1). We searched MEDLINE (OVID), EMBASE (OVID), CENTRAL (OVID), and CINAHL (EBSCO) from inception until October 23, 2023. We also searched the Study Within A Trial (SWAT) repository [14] and the Online Resource for Research in Clinical trials (ORRCA) database [15] for methodological research on recruitment and retention. Finally, reference lists and forward citations of included studies were screened for additional papers.

### 2.2 Eligibility criteria

Eligible studies were randomised trials of melanoma surveillance in people at increased risk due to personal history (e.g., melanoma, transplant, dysplastic naevus syndrome), family history, or as determined by a risk assessment tool or clinical judgment. There were no restrictions by year of publication or language. Detailed criteria are provided in Supplementary table 1.

### 2.3 Study selection

After a pilot test of 50 records, two reviewers (DA and DD) independently screened titles and abstracts. Full texts of potentially relevant papers were assessed by two reviewers (DA and KBr) and reasons for exclusion documented (Supplementary Table 2). Disagreements were resolved through discussion or with a third reviewer (KJLB). A flow chart guided the full-text screening process (Supplementary Fig. 2).

### 2.4 Data extraction and analysis

Data were extracted by DA from the included studies using a tool developed by the reviewers, and a convenience sample (n = 5) was checked by a second reviewer (KJLB). Earlier publications, supplementary materials, and protocols were also reviewed if available. Extracted data included study characteristics, recruitment and retention metrics and strategies to improve these. The following proportion calculations (defined in Table 1) were undertaken: (i) screening-to-eligible, (ii) eligible-to-randomised, (iii) recruitment numbers per month, (iv) response to Patient-Reported Outcome Measures (PROMs). Recruitment and retention strategies were summarised descriptively and grouped thematically based on the ORCCA recruitment and retention domains [15] and participant flow through the study.

**Table 1:** Recruitment data in 21 trials of people at increased risk of melanoma.

Study ID	Recruitment setting	Target	Screened	Eligible (Screened to eligible rate %)	Randomised (Eligible to randomised rate %)	Reasons for non-participation if eligible	Recruitment dates (months)	Recruitment rate (patients recruited/mth)
Ackermann 2022 [25]	2 dermatology clinics and 1 general practice skin clinic	100	481	326 (68)	100 (31)	47 Declined to participate;	November 1, 2018 to May 24, 2019 (7 months)	15
						179 Reasons for nonparticipation unknown		
Bowen 2015 [17]	2 cancer databases: Northwest Cancer Genetics Network, SEER registry at Fred Hutchinson Cancer Research Center	NR	1380	480 (35)	311 (65)	372 - Refused to participate; 74 - Too busy/no time; 63 - Not interested; 47 - Did not want family contact; 42 - Family did not want to participate; 7 - Refuses to give a reason; 15 - Too sick	Late 2005 to mid-2007 (21 months)	15
Bowen 2019 [18]	2 cancer databases as per Bowen 2015	NR	1248	398 (32)	313 (79)	117 - Refused to participate; 25 - Too busy/no time; 29 - Not interested; 22 - Refuses to give a reason.	Late 2005 to mid-2007 (21 months)	15
Geller 2006 [19]	4 dermatology clinics	NR	667	667 (100)	494 (74)	Not reported	October 1998–Dec 2000 (27 months)	18
Geller 2023 [24]	Childhood cancer study (participants initially recruited by 27 clinical sites)	801	1353	1204 (89)	728 (60)	81 - Declined; 381 - Nonresponder; 2 - Deceased	NR	
Glanz 2010 [38]	2 general practice sites	NR	2038	1371 (67)	724 (53)	Not reported	April 2000–May 2001 (14 months)	52
Glanz 2015 [39]	1 general practice	NR	630	221 (35)	206 (93)	5 - Refused; 10 - Unreachable	May 2011–July 2011 (3 months)	69
Manahan 2015 [35]	Cohort study, volunteers	50	559	533 (95)	58 (first of 116 who expressed interest to continue)	10 - Declined to participate	NR	
Manne 2010 [20]	3 Cancer clinics	NR	2382	1448 (61)	443 (31)	483 - Unable to contact; 482 - Refused	February 2006 to September 2008 (32 months)	14
Manne 2021 [27]	3 Dermatology/cancer clinics and cancer database.	420	1411	1261 (89)	441 (35)	776 - Declined to participate; 299 - Not interested; 347 - No reason; 72 – too busy; 23 – Too ill.	October 2019 to March 2020 (6 months)	74
Manne 2022 [34]	1 High risk melanoma clinic	104	299	298 (100)	116 (39)	178 - Declined to participate; 7 - Not interested; 166 - No reason; 4 – too busy;	March 2017–August 2018 (18 months)	6
Marce 2022 [21]	9 hospital centres	900	NR	336	280 (83)	Privacy concerns, uncontactable	December 13, 2017 to April 26, 2019 (17 months)	17
Marek 2018 [36]	1 pigmented lesion clinic	70	119	119 (100)	69 (58)	38 - Did not provide consent (privacy/security concerns, tech savviness, routine change, research aversion); 12 - Device incompatibility	August 2015–January 2017 (18 months)	4
Moncrieff 2022 [16]	Dermatology outpatients – 6 Netherlands hospitals, plus UK sites	180	534	420 (79)	207 (49)	239 - Refused to participate; 156 – transportation issues; 34 – anxiety; 11 – returned to referrer; 38 – no reason.	January 31, 2006, and January 8, 2016 (120 months)	2
Murchie 2022 [26]	2 hospitals (team meeting lists, pathology registers, follow up clinic registers)	240	361	264 (73)	240 (91)	19 - Declined to participate; 4 - Other reasons	January 24, 2018 and March 8, 2019 (14 months)	18
Oliveria 2004 [37]	1 Dermatology clinic	NR	105	105 (100)	100 (95)	Scheduling conflicts and fears about traveling into New York post 9/11	March 2000 for 2 years (24 months)	4
Robinson 2007 [28]	Hospital database	NR	682	540 (79)	130 (24)	22 - Refused; 384 - No Response to Invitation	NR	
Robinson 2010 [29]	Not reported	NR	NR	NR	40	Not reported	NR	
Robinson 2016 [22]	Hospital database, volunteers	430	1481	856 (58)	494 (58)	362 - Declined to participate: 220 - too busy; 81 - unspecified, 61 – wants monitoring only by physician	June 6, 2011, to April 14, 2013 (22 months)	22
Robinson 2020 [23]	Hospital database, previous trial participants.	384	Total not reported	Total not reported	Total 341	Phase 2: 42 – Declined to participate	June 2016 to April 2017 (11 months), participants in Phase 1 were invited to enrol in Phase 2	
			Phase 2450	Phase 2211	Phase 2169 (80)		Recruitment dates for new participants not reported	
Walter 2020 [30]	12 General practices	200	1729	410 (24)	238 (58)	1319 - Did not meet inclusion criteria; 172 - Declined to participate	August 22, 2016–January 6, 2017 (5 months)	53

1. Screening to eligibility proportion: Proportion of screened participants who met the eligibility criteria.

2. Eligibility to randomisation proportion: Proportion of eligible participants who were randomised into the study.

3. Recruitment numbers per month: The recruitment per month was calculated by dividing the total number of participants recruited by the recruitment period in months. For specific start and end dates, exact dates were used. If only months were specified without exact days, the first day of the month was assumed for the start date and the last day of the month for the end date. For qualitative descriptions (e.g., "Late 2005 to mid-2007"), "late" was interpreted as October of the starting year, and "mid" as June of the ending year.

## 3 Results

### 3.1 Search results

Database searches identified 1746 records (Supplementary Fig. 3). After deduplication and title and abstract screening, 82 papers were selected for full-text review. Of these, 54 were excluded (reasons detailed in Supplementary Table 2). Searches of the SWAT repository, ORCCA database and citations yielded no additional references. Finally, 21 trials, reported across 28 papers, were included in the review.

### 3.2 Characteristics of included studies

Detailed characteristics of included trials are provided in Supplementary Table 3. Studies were from the USA (n = 15), Europe (n = 4) and Australia (n = 2), ranged in size from 40 to 728 participants and in duration from 3 to 24 months. Trial populations included melanoma survivors (n = 9), first degree relatives of melanoma survivors (n = 4) and patients assessed as high risk (n = 8) including childhood cancer survivors, those with dysplastic nevus syndrome, or those assessed by a risk assessment tool or clinical judgement. Although most interventions aimed to increase frequency of skin self-examination (SSE) or clinician examination, one study aimed to decrease the frequency of clinical surveillance visits [16]. While most studies allocated participants to groups as individuals, five used family-level allocations [[17], [18], [19], [20], [21]] and two enrolled dyads, which included a high-risk individual and a skin check partner [22,23]. Outcomes included patient-reported metrics of SSE performance (frequency, thoroughness, number of body parts examined, adherence to SSE recommendations), clinician examination frequency [[19], [20], [21], [22],^24^], clinical outcomes (biopsies, new primary and recurrent melanoma diagnoses, survival) [16,22,23,25,26], and psychological outcomes (SSE attitudes, self-efficacy, quality of life, fear of cancer recurrence, anxiety and depression scores) [16,23,[25], [26], [27], [28], [29], [30]].

### 3.3 Recruitment and retention metrics

The trial recruitment characteristics are presented in Table 1. Separate recruitment reports were published by Bowen 2015 and 2019 [31], Manne 2021 [32] and Robinson 2016 [33]. Twelve studies (57 %) reported target sample sizes, including all studies published since 2020 (n = 9). Justifications for sample size calculations were provided in eight studies, including specifications of the expected effect size (based on prior literature or pilot data), statistical power and significance level (e.g., 80 % power, 0.05 alpha), and adjustments for anticipated dropouts. Three studies (14 %) did not meet their recruitment goals. Marce 2022, a cluster RCT targeting first-degree relatives (FDRs) of melanoma patients, aimed to recruit 900 FDRs but enrolled only 280 [21]. This shortfall was due to an overestimation of the average number of FDRs per melanoma patient (anticipated to be 4 but was 2.6). The design of the trial prevented extending the inclusion period to adjust the sample size. Robinson 2020 targeted 384 dyads (participants plus skin check partner) and recruited 341 [23]. The study noted that participants more readily enrolled in the remote training phase than in the in-person training phase. The primary barrier to eligibility was the absence of a skin check partner, necessary for enrolment as dyads. Geller 2023, with a target of 801, enrolled 728 participants, but did not discuss the reasons for not meeting the recruitment goal [24].

The mean screened-to-eligible percentage across the 18 trials with sufficient data, was 75 % (range 24%–100 %). Higher percentages were associated with narrowly defined screening populations that closely aligned with the eligibility criteria [34]. Conversely, broader screening approaches (e.g. general practice settings), had lower proportions found to be eligible. The mean eligible-to-randomised percentage in the same 18 trials was 63 % (range 24%–95 %). High risk dermatology clinics had higher proportions randomised, possibly due to more targeted and motivated populations in these clinical settings. Eighteen trials (86 %) reported reasons for non-participation among eligible participants; 13 cited broad reasons such as "declined to participate" or "not interested," while five presented specific explanations, including anxiety, privacy issues, and logistical challenges like transportation difficulties and time constraints. Mean recruitment per month was 25 participants (range 2–74) with the duration of recruitment ranging from three to 120 months (10 years).

Trial retention and response characteristics are presented in Table 2. The mean percentage of participants responding to surveys at the primary timepoint across 19 trials reporting patient-reported outcomes was 85 % (range 59%–100 %). Withdrawals (range 1%–28 %) were reported in eight studies. Differential losses, where intervention groups experienced higher losses than control groups, were noted in four studies [22,26,27,34]. Most studies did not report reasons for participant loss. However, specific reasons were provided in Ackermann 2022 (lack of time, did not wish to continue) and Manahan 2015 (moved away, preferred own doctor, lack of time, technical issues).

**Table 2:** Retention and response data for patient reported outcomes in 21 trials of people at increased risk of melanoma.

Study ID	Randomised	Reporting	Withdrawals (%) Loss to follow up (%)	PROM Primary timepoint	Response T1	Reasons for Withdrawal or Non-Response	Differential losses
**Ackermann 2022**	100	Withdrawals, loss to follow up, response for each outcome and timepoint	19 (19)	6 months	66 (66)	Lack of time, did not wish to continue	No
			10 (10)				
**Bowen 2015**	311	Response to 12 month survey.		12 months	277 (89)	Not reported	Not reported
**Bowen 2019**	313	Response to 12 month survey		12 months	278 (89)	Not reported	No
**Geller 2006**	494	Response to survey at each timepoint		12 months	314 (64)	Not reported	No
**Geller 2023**	728	Withdrawals, loss to follow up, response to survey at each timepoint	14 (2)	18 months	655 (90)	Not reported	No
			59 (8)				
**Glanz 2010**	724	Response to telephone interview assessment and 4 month survey		4 months	596 (82)	Not reported	No
**Glanz 2015**	206	Response to 3 month survey		3 months	189 (92)	Not reported	No
**Manahan 2015**	58	Withdrawals	9 (16)	Nil		Moved away, preferred own doctor, lack of time, technical issues	No
**Manne 2010**	443	Response to 12 month survey		12 months	384 (87)	Not reported	No
**Manne 2021**	441	Withdrawals, response to survey at 8 week, 24 week, 48 week	5 (1)	48 weeks	382 (87)	Not reported	Yes – mySmartskin 42/224 not complete, usual care 14/217 not completed
**Manne 2022**	116	Survey response		13 weeks	99/116	Not reported	Yes Intervention loss 13/56, usual care 4/60
**Marce 2022**	280	Phone call by research assistant data collection		12 months	280 (100)	No losses	n
**Marek 2018**	69	Survey response		6 months	69 (100)	No losses	
**Moncrieff 2022**	388	Loss to follow up, withdrawals, clinical events, PROMs not returned.	28 (7)	5 years	240)	Recurrences, deaths	No
**Murchie** **2022**	240	Loss to follow up, withdrawals, survey response at 3, 6, 12 months	9 (4)	12 months	168 (70)	Yes, broad reasons	Withdrawals all in intervention group Patient questionnaires returned at each timepoint slightly less in intervention group
**Oliveria 2004**	100	Survey response at 4 months		4 months	85 (85)	Not reported	No
**Robinson 2007**	130	Survey response at 4 months		4 months	130 (100)	No losses	
**Robinson 2010**	40	No losses reported		4 months	Not reported	Not reported	
**Robinson 2016**	494	Survey response at 4, 12, 48 months		24 months	291 (59)	Not reported	Yes – less losses in control vs intervention groups
**Robinson 2020**	341	“drops” response at 9 months, 18 months	16 (5)	18 months	303 (89)	No	no
**Walter 2020**	238	Loss to follow up, survey response 12 months	16 (7)	12 months	157 (86)	No	no

The response to PROMs was calculated as the number of participants who completed the PROM divided by the number randomised. This calculation was done at the primary timepoint.

### 3.4 Strategies

#### 3.4.1 Recruitment

**Fig. 1:**
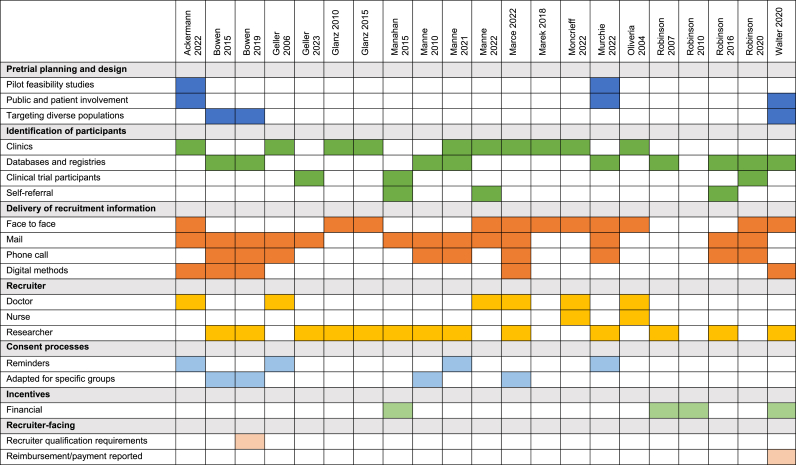
Recruitment strategies identified in 21 trials of people at increased risk of melanoma.

Commonly identified recruitment strategies are summarised in Fig. 1 and Table 3, with details per study in Supplementary Table 3.

**Table 3:** Summary of recruitment strategies.

Category	Strategy Details	Example Studies and Details
**Pre-trial Planning and Design**	**Trial design considerations**: sample size calculations and defining eligibility criteria to optimise recruitment efficacy.	**Manne 2021** - Detailed sample size calculations considering both primary and secondary outcomes. **Marek 2018** - Targeted enrolment based on prior literature. **Robinson 2016, Robinson 2020** - Sample size rationale and estimation included.
	**Patient and public involvement**	**Ackermann 2022, Walter 2020** - Public and patient involvement representatives provided input on the study design and materials. **Murchie 2022** - Co-design of the intervention with potential recipients. **Bowen 2015, Bowen 2019, Manne 2021, Manne 2022, Murchie 2022, Walter 2020** – Pre-trial usability testing of the intervention.
	**Eligibility screening**: pre-trial screening questionnaires to determine participant suitability.	**Glanz 2010** - Patients given brief screening questionnaire in the waiting room. **Manahan 2018** required participants to complete a pre-enrolment eligibility questionnaire on teledermatology acceptance
	**Pilot studies**: conducting pilot studies to test feasibility and refine the recruitment process.	**Ackermann 2022, Murchie 2022** - Pilot study testing feasibility of larger scale study. **Murchie 2022**
	**Recruiting diverse populations**	**Walter 2020 -** Protocol specifies recruitment at different times of day and days of week, to approach people of different ages, gender and educational level. **Bowen 2012** noted that despite vigorous efforts, unable to recruit lower income families as frequently.
	**Adaptations for specific groups**: tailoring recruitment processes for families or specific patient groups such as those identified from cancer registries.	**Bowen 2015, Bowen 2019, Manne 2010, Marce 2022** – adapted processes to recruit families.
**Identification of Participants**	**Databases and registries**: using electronic health records, cancer databases, and hospital databases to identify potential participants.	**Bowen 2015, Bowen 2019** - Use of electronic health records to identify participants. **Manne 2010** - Participants identified from 3 cancer clinic registries. **Robinson 2016** - Patients identified by electronic medical records of the Northwestern Medicine health care system. **Robinson 2020** - Participants identified through the Enterprise Data Warehouse (EDW) of Northwestern University.
	**Direct engagement in clinics**: recruiting participants directly at clinics during routine or follow-up visits.	**Ackermann 2022** - Participants identified during regular follow-ups by nine physicians from two melanoma specialty clinics and one primary care skin cancer clinic. **Glanz 2010, Glanz 2015** - Recruitment in the waiting room of primary care practices. **Manne 2022** - Patients informed during a clinic visit by the dermatologist. **Moncrieff 2022** - Recruitment at clinic follow-up visits. **Oliveria 2004** - Recruited during scheduled clinic visits. **Walter 2020** - Opportunistic recruitment in the reception area of general practices.
	**Self-referral**	**Manahan 2015 –** volunteers. **Manne 2022** - Participants could self-refer after learning about the study at www.clinicaltrials.gov**. Robinson 2016 –** newspaper advertisements
**Delivery of Information**	**Method of information delivery**: various methods such as face-to-face, mail followed by phone calls, and direct mailing of detailed information and consent forms.	**Ackermann 2022** - Information delivered in person or by mail. **Bowen 2015, Bowen 2019** - Mail followed by phone call. **Geller 2006** - Mail with follow-up calls. **Geller 2023** - Mail. **Glanz 2010** - Face-to-face in the waiting room. **Glanz 2015** - Face-to-face in the waiting room. **Manne 2010** - Mail followed by phone call. **Manne 2021** - Mail followed by phone call. **Manne 2022** - Face-to-face in clinic or mailed for self-referrals. **Marce 2022** - Face-to-face for index cases; telephone, email, or mail for FDRs. **Marek 2018** - Face-to-face. **Moncrieff 2022** - Face-to-face. **Murchie 2022** - Mail followed by choice of phone call or face-to-face visit. **Oliveria 2004** - Face-to-face. **Robinson 2007** - Not reported. **Robinson 2010** - Not reported. **Robinson 2016** - Mail followed by phone call. **Robinson 2020** - Mixed: face-to-face, letter, or telephone call. **Walter 2020** - Face-to-face.
	**Personnel involved**: information delivered by physicians, nurse practitioners, or specialised study staff.	**Ackermann 2022** - Information delivered by treating clinicians. **Glanz 2010, Glanz 2015** - Information delivered by study staff. **Moncrieff 2022** - Information delivered by physicians or nurse practitioners. **Oliveria 2004** - Information delivered by physicians or nurses.
**Consent Process**	**Reminders**	**Ackermann 2022** - Research staff contacted participants via phone if no response within two weeks. **Geller 2006** - Non-respondents received a second mailing and a minimum of five follow-up calls. **Manne 2021** - Followed up via telephone after initial mailing. **Murchie 2022** - Non-respondents contacted by phone or face-to-face.
	**Opt in/opt out options**	**Ackermann 2022** - Participants could decline participation by returning the card, phone, or email. **Bowen 2015, Bowen 2019** - Opt-out options after each step of mailing information to physicians, cases, and FDRs prior to phone call to confirm eligibility.
	**Financial incentives or vouchers**	**Manahan 2015** - Participants reimbursed with a $100(AU) voucher. **Walter 2020** - Participants reimbursed with a voucher for £10. **Robinson 2007, 2010** - Participants offered nominal payment.
**Incentives**	**Recruiter qualification requirements**	**Geller 2006** - Dermatologists had to meet criteria to participate.
**Recruiter-facing**	**Reimbursement or incentives for recruitment support**	**Walter 2020** - General practices reimbursed to cover administrative or time costs associated with supporting the study.

##### 3.4.1.1 Trial design and pre-trial planning

Two trials reported incorporating public and patient involvement in study design and materials [25,30], while another trial reported co-design of the intervention with potential recipients [26]. Six trials conducted pre-trial user testing to establish acceptability and refine interventions [17,18,26,27,30,34]. Additionally, one study conducted a usability survey with the first 15 pairs of participants to gather immediate feedback. These pairs experienced technical issues with the study tablets, such as image loading delays and chapter loading failures, which were resolved [22]. Two pilot studies tested the feasibility of larger-scale studies including Ackermann 2022 which reported the proportion of eligible and contacted patients who were randomised as the primary outcome [25]. Manne 2021 reported flexible recruitment methods tailored to different sites to enhance efficiency and participant enrolment. Manahan 2018 targeted recruitment efficiency through over-screening with 559 potential participants to achieve a sample size of 50. Additionally, the study required participants to complete a pre-enrolment eligibility questionnaire on teledermatology acceptance to enhance recruitment efficiency. Two studies described specific efforts to recruit a diverse demographic. Walter 2020 varied recruitment times throughout the day and week to target participants from diverse age groups, genders, and educational levels, while Bowen 2012 described challenges in recruiting lower-income families despite repeated, varied communication efforts [31].

Trials recruiting families of index case melanoma patients implemented several modifications to identification processes to efficiently recruit both patients and their first-degree relatives. These modifications included using electronic health records to identify index cases, asking dermatologists to approach the index cases to obtain permission to contact their FDRs, using various communication methods, such as phone, email, voicemail, face-to-face, and written contact, to find and engage FDRs and then allocating participants to intervention groups as a family unit. In addition, some studies involving participants from cancer registries contacted treating physicians before approaching patients to ensure appropriateness.

##### 3.4.1.2 Identification of participants

All studies identified participants using strategies that directly targeted specific individuals, such as electronic databases, identification at scheduled visits, and previous trial participants drawn from clinical populations. Three studies additionally used broader methods to reach participants, including newspaper advertisements [22], clinicaltrials.gov referrals [34], and word of mouth [35]. Five studies recruited participants at dermatology or high-risk melanoma clinic visits [16,25,34,36,37], and three at general practice clinics [30,38,39]. Nine studies used clinic databases, cancer registries, or hospital databases to identify participants. Robertson 2020 used the Enterprise Data Warehouse (EDW) of Northwestern University, a repository of patients willing to participate in research. Additionally, three studies recruited participants from previous trials [22,23,35].

##### 3.4.1.3 Delivery of information

Several studies used a range of communication methods to reach potential participants, including phone, email, voicemail, face-to-face meetings, and written contact. Initial methods for delivering information to potential participants in clinical trials included face-to-face interactions, mail followed by phone calls, and direct mailing of detailed information and consent forms. Face-to-face information was typically delivered by physicians, nurse practitioners, or research staff.

##### 3.4.1.4 Consent process

Consent was typically obtained face to face, but also acquired remotely via mail, phone, and electronically via an online system. Reminders to complete the remote consent process were reported in several studies. Ackermann 2022 specified that if there was no response within two weeks, research staff would contact participants via phone. Geller 2006 sent a second mailing and made at least five follow-up calls to non-respondents. Manne 2021 arranged for a second mailing when necessary. Robinson 2016 and 2020 used multiple follow-up methods, including phone and letter, to contact participants. Bowen 2015 and 2019 reported a range of communication methods such as phone, email, voicemail, face-to-face contact, and written communications to engage potential participants. Most studies required participants to actively opt in, typically by returning a signed consent form. However, Bowen 2015 and 2019 offered participants options to opt-out at multiple stages of the study and in Marce 2022, the process involved contacting first-degree relatives if “non-opposition” was expressed by the index case.

##### 3.4.1.5 Incentives

Financial incentives were offered in four studies. Manahan 2015 offered a (US) $100 voucher, Walter 2020 provided £10 reimbursement, and Robinson 2007 and 2010 awarded “nominal” payments to participants.

##### 3.4.1.6 Recruiter-facing strategies

Few recruiter facing strategies were reported. In Geller 2006, recruiting dermatologists were selected based on specific criteria, such as managing at least 25 invasive melanoma cases per year. Additionally, Walter 2020 reported that participating general practices received reimbursements to cover the administrative or time costs associated with supporting the study.

##### 3.4.1.7 Recruitment methods publications

No studies reported an evaluation of their recruitment strategies. There were three separately published descriptions of recruitment processes. One observational cross-sectional study included 368 melanoma patients who either enrolled in (n = 187) or declined (n = 181) a partner-assisted SSE intervention (Robertson 2016). No significant age or gender differences were observed between groups. Enrolled participants had higher perceptions of melanoma risk, severity, and early detection benefits. The main reasons for declining were being too busy (males) and lack of partner support (females) [22,33]. Bowen 2012 described recruitment for a web-based intervention for melanoma-affected families. Recruitment included the melanoma case, a first-degree relative (FDR), and an additional family member who is a parent of a child under 18. Recruitment involved three steps: physician consent, index case consent and contacting family members. Higher educational attainment predicted participation for both cases and FDRs. FDRs were more likely to enrol if female. Parents were more likely to enrol if the case was recently diagnosed or lived in the same city [17,18,31]. Manne 2021 published a protocol with additional information about intervention development and recruitment processes and metrics [32]. They note that the study predominantly included non-Hispanic whites with higher socioeconomic status, highlighting the need for more diverse recruitment in future research.

#### 3.4.2 Retention strategies

**Fig. 2:**
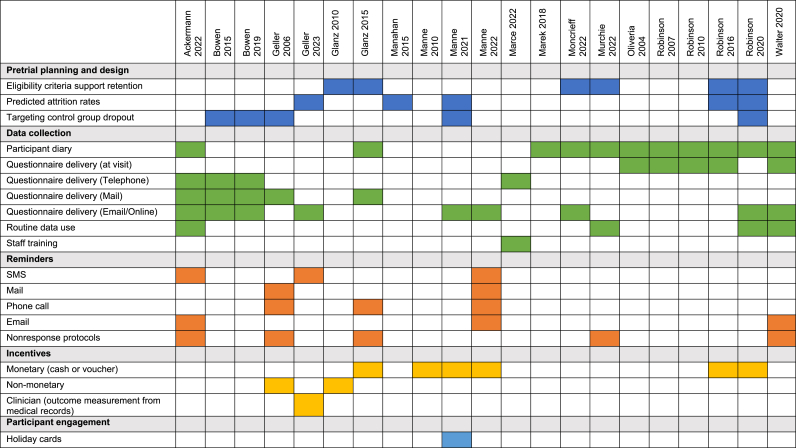
Retention and response strategies in 21 trials of people at increased risk of melanoma.

Commonly identified retention strategies are summarised in Fig. 2 and Table 4 and detailed per study in Supplementary Table 3.

**Table 4:** Summary of retention and response strategies.

Category	Strategy Details	Example Studies and Details
**Pretrial Planning and Study Design**	**Exclusion Criteria**	**Glanz 2010 & 2015** - Participants excluded if planning long travels during study. **Moncrieff 2022** and **Murchie 2022** - Exclusion of those unable to complete questionnaires. **Robinson 2016** – Excludes those unable to commit to skin exams by study dermatologist every 4 months for 24 months. **Robinson 2020** - Excludes those unable to commit to completing surveys at specified intervals.
	**Sample Size Consideration**	**Geller 2023, Manahan 2015, Manne 2021, Robinson 2016, Robinson 2020** - Included predicted attrition rates of 10%–25 % in sample size calculations.
	**Intervention Preference**	**Bowen 2015, Bowen 2019** - Screening to confirm no treatment preference before assignment. Immediate offer to complete questionnaire. **Bowen 2015, Bowen 2019, Geller 2006, Manne 2021, Robinson 2020 -** Delayed intervention: control group receives access to the intervention at trial completion.
**Data Collection**	**Questionnaire Delivery**	**Ackermann 2022** - Delivery via mail or online link. **Geller 2006** - Mailed baseline questionnaires with repeat mailings. **Walter 2020** - Baseline questionnaire in person, follow-ups online. **Moncrieff 2022** - Mailed questionnaires with pre-stamped return envelopes.
	**Participant Diary**	**Ackermann 2022 –** monthly health resource diary
	**Routine Data Use**	**Walter 2020** - Audit of GP records used for routine data. **Murchie 2022** - Collection of routine data from health records.
	**Centralisation and Standardisation**	**Marce 2022** - Phone interviews centralised to standardise outcome assessments.
	**Training for Staff**	**Marce 2022** - Clinical research assistant trained for data collection.
**Reminders**	**Mode**	**Ackermann 2022** - Up to five reminders by SMS or email. **Manne 2022** - Automated email, telephone, text message, and mail reminders. **Murchie 2022** - Email, SMS, phone calls for non-responders.
	**Nonresponse Protocols**	**Ackermann 2022** - Telephone administration offered for persistent nonresponse. **Murchie 2022** - Direct tracing of participants using preferred methods for prompts or indirectly by contacting the GP.
**Incentives**	**Participant**	**Geller 2006** - Movie tickets or gift cards upon survey completion. **Glanz 2010** - Small gifts like magnets, pens. **Glanz 2015** ($20 USD), **Robinson 2016** ($20 USD), **Robinson 2020** ($20 to $25 USD), **Manne 2010** ($10 USD), **Manne 2021** ($25 USD), and **Manne 2022** ($20 USD) payment per survey completed
	**Physician**	**Geller 2023 -** Physician offices received $25.00 for this verification
**Participant Engagement**	**Building Community**	**Manne 2021** - Sending birthday and non-denominational holiday cards.

##### 3.4.2.1 Pretrial planning and trial design

In pretrial planning and study design, several strategies were used to ensure participant retention. Exclusion criteria were established to maximise ongoing participation: Glanz 2010 and Glanz 2015 excluded participants planning travel during the study period; Murchie 2022 excluded those unable to complete questionnaires; and Robinson 2020 excluded participants who could not commit to completing surveys at specified intervals. Anticipated attrition rates (range 10%–25 %) were included in the sample size calculations in five studies. To minimise potential bias and dropout due to treatment preferences, screening calls were conducted in Bowen 2015 and Bowen 2019 to ensure participants had no preference between intervention and control groups. Additionally, five trials implemented a delayed intervention strategy, offering the control group access to the intervention at the end of the trial to sustain engagement and reduce dropout rates.

##### 3.4.2.2 Data collection

In older trials, questionnaires were administered by mail (n = 5), phone (n = 4), or face-to-face at clinic visits (n = 5), while more recent studies used email or online platforms (n = 9). Pre-stamped return envelopes were included with mailed questionnaires to support response. To achieve consistency across different sites, centralisation and standardisation of data collection were implemented, as seen in Marce 2022 where phone interviews were centralised to standardise outcome assessments [21]. Additionally, Marce 2022 provided training for clinical research assistants to ensure consistent data collection practices. Data collection was supported by incorporating routinely collected health data, including clinical information such as new melanoma diagnoses [23,25,26] and GP attendance [30] which helped to avoid issues with missing data. Health records were also used to verify patient responses [21,24].

##### 3.4.2.3 Reminders

Seven studies reported reminder strategies to enhance response rates, using protocols with varying numbers of reminders, modes of communication, and intervals between reminders. Ackermann 2022 sent up to five reminders via SMS or email if the online questionnaire was not completed within a week. For persistent non-response, researchers offered phone administration. Geller 2006 used a follow-up protocol that included a repeat mailing and up to five phone calls. Glanz 2015 conducted phone interviews if mailed surveys were not returned after two reminders. Murchie 2022 sent reminders to non-responders after three weeks, Walter 2020 sent up to two reminders via email and Manne 2022 reported using automated email, phone, text message, and mail prompts to encourage participants to complete online surveys.

##### 3.4.2.4 Incentives

Eight studies reported using participant incentives including cash, vouchers, and non-monetary incentives. Geller 2006 provided movie tickets or gift cards for survey completion, and Glanz 2010 used small gifts like magnets and pens as incentives. Studies offering payment (all USD) per survey completed included Glanz 2015 ($20), Robinson 2016 ($20), Robinson 2020 ($20 to $25), Manne 2010 ($10), Manne 2021 ($25), and Manne 2022 ($20). Additionally, physician offices received a $25 payment per participant for medical record review in Geller 2023.

##### 3.4.2.5 Participant engagement

Participant engagement strategies included sending birthday and holiday cards (Manne 2021).

## 4 Discussion

This review of 21 RCTs of melanoma surveillance suggests that the CONSORT statement has improved reporting of recruitment metrics, with all trials published since 2020 reporting target sample size and including a participant flow diagram. However, reporting on recruitment and retention metrics and strategies remains suboptimal even in recent trials. Consistent reporting of recruitment, retention, and response metrics is essential for guiding future research design and may also provide insights into the potential uptake of interventions in broader clinical settings.

Recruitment challenges were evident (often indirectly reported) across several of the included trials: three out of twelve failed to meet their target sample sizes, one trial took 10 years to recruit despite a high proportion of eligible participants, and recruitment rates varied significantly (range 2–74 participants per month). These challenges emphasise the need for evidence-based recruitment strategies. The mean screened-to-eligible proportion was 75 %, with higher proportions in narrowly defined populations, potentially improving trial success but limiting broader clinical applicability of findings. Broader screening populations, such as general practice settings had lower screened-to-eligible proportions, potentially increasing recruitment times and costs but enhancing generalisability. To address these contextual differences, tailored strategies are needed. High-risk populations may benefit from targeted outreach through high-risk clinics, specialist involvement, and integration into established care pathways to maximise efficiency, while lower-risk groups may require broader outreach via general practitioners, advertisements, and high-risk identification tools (e.g., BRAT or Melatools) to refine the broader population.

A substantial proportion of eligible participants (37 %) chose not to proceed with the trial, with detailed reasons for non-enrolment documented in only five (24 %) trials. A higher eligible-to-randomised proportion may signal greater potential acceptance of the intervention in clinical practice, while a lower proportion could indicate barriers that limit uptake, such as treatment complexity, lack of perceived benefit, or concerns about side effects. Considering both the screened-to-eligible and eligible-to-randomised proportions together could indicate potential uptake in clinical practice. However, pre-screening selection processes and factors that enhance participation in research settings (financial incentives and increased support) are likely to bias estimates resulting in overestimation of actual uptake in practice. In addition, these metrics are likely to vary widely depending on the specific context of the study, including the disease area, the complexity of the intervention, and the participant population.

Response rates also varied widely. While missing data below 5 % is often considered small and over 20 % large, the Cochrane Handbook cautions that no universal threshold exists, as the impact depends on the outcome type and event risk [40,41]. Trials of shorter duration and face-to-face questionnaire completion reported higher responses. Differential losses where intervention groups experienced higher attrition than control groups occurred in four studies suggesting that specific aspects of the interventions may have adversely affected participant retention. To ensure sufficient power, incorporating anticipated attrition rates into sample size calculations, as demonstrated by five studies, is recommended.

The CONSORT flow diagram requires authors to report numbers for those "lost to follow-up" and those who "discontinued intervention," including reasons [42]. However terms like withdrawals, loss to follow-up, and response (which may be at different timepoints or per questionnaire) were used interchangeably in the included papers without clear distinctions, complicating data extraction and interpretation. Participants may also remain in the study but not provide outcome data or they may withdraw but permit the passive use of their routinely collected data. Expanding the CONSORT checklist and flow diagram to explicitly include missing data per outcome and timepoint would enhance transparency and facilitate interpretation. While the CONSORT checklist (item 16) requires reporting the number of participants included in each analysis (denominator), this is not explicitly represented in the flow diagram [42]. Adding these details, as recommended by the CONSORT extension for pilot studies, would improve clarity. These data would also be useful for applying the ROB2 risk of bias tool (Domain 3: Bias Due to Missing Outcome Data directly addresses retention by examining the completeness of data per outcome) [43].

The identified recruitment strategies align with ORCCA recruitment categories (design, pre-trial planning, trial conduct changes, modifications to the consent process, modification to information given to potential participants, recruiter-facing, and incentives), with notable gaps in trial conduct changes and recruiter-facing interventions. Public and patient involvement was limited, though some trials incorporated user testing to refine interventions. Financial incentives and post-trial access to interventions were used to enhance recruitment. Key predictors of participation included higher educational attainment, recent diagnosis, and female gender. Only two studies explicitly aimed to recruit diverse populations, including people at higher risk of adverse outcomes who could benefit more from early detection interventions. Strategies were generally applied on an ad hoc basis and not well-described and lacked evaluations of effectiveness making it difficult to form recommendations. Evidence for specific recruitment strategies is limited. A 2018 Cochrane review found high-certainty evidence that unblinded trials (all trials in review) and phone reminders improved recruitment by 10 % and 6 %, respectively [7]. Other strategies (including financial incentives and changes to how potential participants receive information) provided moderate to very low-certainty evidence, indicating a need for further replication and research.

Retention strategies were less frequently reported, but also align with the ORRCA categories (data collection, study design, and participants), with notable gaps in reporting strategies addressing sites, staff, and central study management. Key strategies included limiting eligibility to those likely to complete participation activities, using incentives like cash and non-monetary gifts and offering the control group access to the intervention at the end of the study. Data collection methods included participant diaries, mailed questionnaires, phone calls, face-to-face administration, with more recent trials moving to online platforms. Reminder strategies varied widely, with no consistency in the number of reminders, modes of communication, or intervals between reminders. Similar strategies have been used in clinical trials more broadly. A systematic mapping review of retention in 80 NIHR Health Technology Assessment trials conducted from January 2020 to June 2022 reported strategies including flexibility in data collection methods (53 %), the use of participant diaries (38 %), routine data use (29 %), patient input (26 %), phone reminders (26 %), postal reminders (25 %), pre-paid return postage (18 %), prioritisation of key outcome collection (15 %), and participant newsletters (15 %) [44]. However, a 2022 Cochrane review found no high-certainty evidence for effective retention strategies; most strategies in use have low, very low or no evidence to support their effectiveness. Notably, participant diaries, despite their frequent use, have moderate certainty evidence that they may actually reduce retention [10]. This emphasises the need for evidence-based trial processes to avoid using strategies that may be ineffective or even harmful.

This review is the first to explore recruitment and retention in melanoma surveillance trials. Its systematic methodology for trial selection and data collection reduces the potential for bias. The main limitation of this study is its dependence on published data that is disclosed by authors, which was often limited even after reviewing trial protocols and earlier trial publications. Most trials used multiple strategies without evaluation, making it difficult to determine which elements were effective and necessary.

Our results highlight the need for increased transparency in reporting trial recruitment and retention. Developing a standard methodological framework for systematic reporting may be considered, potentially as part of the CONSORT statement. This could involve expanding sections 4b and 14a, updating the flow chart, and incorporating recruitment and retention metrics and strategy descriptions. The TIDieR checklist could also be used to enhance the reporting of complex clinical trial recruitment and retention interventions [43]. Including a checklist in the supplement would provide a comprehensive approach to improve the overall quality and transparency of clinical trials. To strengthen the evidence base on optimal strategies for recruitment and retention, future RCTs should consider incorporating Studies Within A Trials (SWATs) when implementing recruitment and retention strategies. Practical frameworks and examples for designing and embedding SWATs are available through resources like Trial Forge (https://www.trialforge.org/swat-resources/) [45] and the University of York's SWAT repository (https://www.york.ac.uk/healthsciences/research/trials/swats/swatresources/) [14]. Additionally, integrating Public and Patient Involvement into trial design may enhance recruitment and retention by ensuring strategies are patient-centred and acceptable. Priority populations, such as underrepresented or disadvantaged groups, require culturally tailored materials, collaboration with community organisations, and logistical support to address barriers. Collaborating with patient advisory panels and refining materials through pilot testing may further optimise these strategies for diverse groups [46,47]. Future research could also explore cross-learning opportunities from recruitment and retention strategies used in other cancer cohorts to contextualise and extend our findings [48].

## 5 Conclusion

This scoping review describes recruitment and retention within melanoma surveillance trials, revealing substantial variability, poor reporting of strategies and few evaluations. Future studies should prioritise the development of standardised reporting frameworks for recruitment and retention, incorporate SWATs to evaluate strategies, and emphasise inclusive approaches to enhance generalisability and impact.

## Support

KLJB is supported by an NHMRC Investigator Grant (2019/GNT1174523). JH is supported by a Cancer Institute NSW Early Career Fellowship (2020/ECF1158). DA is supported by an NHMRC Postgraduate Scholarship (2014163). MJ is supported NHMRC CRE (2006551) and NHMRC Synergy (2009923)

## CRediT authorship contribution statement

**Deonna M. Ackermann:** Writing – review & editing, Writing – original draft, Visualization, Validation, Project administration, Methodology, Funding acquisition, Formal analysis, Data curation, Conceptualization. **Karen Bracken:** Writing – review & editing, Validation, Supervision, Methodology, Formal analysis, Data curation, Conceptualization. **Jolyn K. Hersch:** Writing – review & editing, Visualization, Supervision, Methodology, Conceptualization. **Monika Janda:** Writing – review & editing, Visualization, Supervision, Methodology, Conceptualization. **Robin M. Turner:** Writing – review & editing, Supervision, Methodology, Conceptualization. **Katy J.L. Bell:** Writing – review & editing, Visualization, Validation, Supervision, Resources, Methodology, Funding acquisition, Formal analysis, Conceptualization.

## Funding

This research project was funded by a National Health and Medical Research Council (NHMRC) Project grant (1163054) and a University of Sydney Research Accelerator (SOAR) Prize. The funder was not involved in study design, data collection, data analysis, manuscript preparation or publication decisions.

## Declaration of competing interest

The authors declare that they have no known competing financial interests or personal relationships that could have appeared to influence the work reported in this paper.

## Data Availability

Data will be made available on request.
