# Supplementary material for: Participant recruitment and retention in randomised controlled trials of melanoma surveillance: A scoping review
Source: Contemp Clin Trials Commun. 2025 Feb 16;44:101461. doi: 10.1016/j.conctc.2025.101461 (PMC11883296; doi:10.1016/j.conctc.2025.101461)
Supplement: Multimedia component 1 [file mmc1.docx]

**Supplementary material contents**

| **Title** | **Page** |
| --- | --- |
| **Supplementary table 1: Scoping Review Research Question (Population, Concept, Context) and eligibility criteria** | **2** |
| **Supplementary table 2 Excluded studies and reasons for exclusion** | **3** |
| **Supplementary table 3: Characteristics of included studies** | **5** |
| **Supplementary table 4: Recruitment and retention strategies by study** | **9** |
| **Supplementary figure 1: MEDLINE Search Strategy** | **14** |
| **Supplementary figure 2: Full text screening flow chart** | **15** |
| **Supplementary figure 3: PRISMA diagram** | **16** |
| **Supplementary methods: PRISMA-ScR checklist** | **17** |

**Supplementary table 1: Scoping Review Research Question (Population, Concept, Context) and eligibility criteria**

| Population | People at increased risk of melanoma due to prior personal medical history (e.g., melanoma, transplant, dysplastic naevus syndrome), family history of melanoma or as determined by a risk assessment tool or clinical judgement. |
| --- | --- |
| Concept | What evidence exists on:   1. Recruitment metrics and strategies for promoting recruitment. 2. Retention metrics and methods for promoting retention and task response. |
| Context | Randomised clinical trials of melanoma surveillance |
| Inclusion criteria | (i) the study design was an RCT, (ii) participants were recruited from a clinical care setting, (iii) participants were at increased risk of melanoma due to personal medical history (e.g., melanoma, transplant, dysplastic naevus syndrome) or family history of melanoma, or as determined by a clinical assessment or risk assessment tool, (iv) an outcome was the early detection of melanoma or a surrogate for this (e.g., improved SSE practice) |
| Exclusion criteria | (i) trial registrations, conference abstracts and protocols of RCTs, without report of the actual RCT findings, (ii) participants not at increased risk of melanoma (e.g., non-selective recruitment from the general population or a general practice population), (iii) participants undergoing current treatment for melanoma, (iv) outcomes restricted to primary prevention e.g., change in sun protection behaviours. |

**Supplementary Table 2: Excluded studies and reasons for exclusion^1^**

| Report | Exclusion reason |
| --- | --- |
| Aneja S, Brimhall AK, Kast DR, et al. Improvement in Patient Performance of Skin Self-examinations After Intervention With Interactive Education and Telecommunication Reminders: A Randomized Controlled Study. Arch Dermatol. 2012;148(11):1266–1272. | Participants not at high risk of melanoma |
| Bowns IR, Collins K, Walters SJ, McDonagh AJ. Telemedicine in dermatology: a randomised controlled trial. Health Technol Assess. 2006 Nov;10(43):iii-iv, ix-xi, 1-39. | Intervention not related to early detection of melanoma |
| Carli P, de Giorgi V, Chiarugi A, et al. Addition of dermoscopy to conventional naked-eye examination in melanoma screening: a randomized study. J Am Acad Dermatol. 2004 May;50(5):683-9. | Outcome not related to the early detection of melanoma |
| Chiu V, Won E, Malik M, Weinstock MA. The use of mole-mapping diagrams to increase skin self-examination accuracy. J Am Acad Dermatol. 2006 Aug;55(2):245-50. | Participants not at high risk of melanoma |
| Glazebrook C, Garrud P, Avery A, at al. Impact of a multimedia intervention "Skinsafe" on patients' knowledge and protective behaviors. Prev Med. 2006 Jun;42(6):449-54. | Intervention not related to early detection of melanoma |
| Hanrahan PF, D'Este CA, Menzies SW, et al. A randomised trial of skin photography as an aid to screening skin lesions in older males. J Med Screen. 2002;9(3):128-32. | Population setting |
| Hanrahan PF, Hersey P, Watson AB, Callaghan TM. The effect of an educational brochure on knowledge and early detection of melanoma. Aust J Public Health. 1995 Jun;19(3):270-4. | Population setting |
| Hay JL, Oliveria SA, Dusza SW, et al. Psychosocial mediators of a nurse intervention to increase skin self-examination in patients at high risk for melanoma. Cancer Epidemiol Biomarkers Prev. 2006 Jun;15(6):1212-6. | Outcome not related to the early detection of melanoma |
| Horsham C, Baade P, Kou K, at al. Optimizing Texting Interventions for Melanoma Prevention and Early Detection: A Latin Square Crossover RCT. Am J Prev Med. 2021 Sep;61(3):348-356. | Population setting |
| Hultgren BA, Turrisi R, Mallett KA, et al. Influence of Quality of Relationship Between Patient With Melanoma and Partner on Partner-Assisted Skin Examination Education: A Randomized Clinical Trial. JAMA Dermatol. 2016 Feb;152(2):184-90. | Outcome not related to the early detection of melanoma |
| Janda M, Baade PD, Youl PH, et al. The skin awareness study: promoting thorough skin self-examination for skin cancer among men 50 years or older. Contemp Clin Trials. 2010 Jan;31(1):119-30. | Population setting |
| Janda M, Horsham C, Vagenas D, et al. Accuracy of mobile digital teledermoscopy for skin self-examinations in adults at high risk of skin cancer: an open-label, randomised controlled trial. Lancet Digit Health. 2020 Mar;2(3):e129-e137. | Population setting |
| Janda M, Neale RE, Youl P, et al. Impact of a video-based intervention to improve the prevalence of skin self-examination in men 50 years or older: the randomized skin awareness trial. Arch Dermatol. 2011 Jul;147(7):799-806. | Population setting |
| Janda M, Youl P, Neale R, et al. Clinical skin examination outcomes after a video-based behavioral intervention: analysis from a randomized clinical trial. JAMA Dermatol. 2014 Apr;150(4):372-9. | Population setting |
| King AJ, Carcioppolo N, Grossman D, et al. A randomised test of printed educational materials about melanoma detection: Varying skin self-examination technique and visual image dose. Health Education Journal. 2015;74(6):732-742. | Participants not at high risk of melanoma |
| Maganty N, Ilyas M, Zhang N, Sharma A. Online, game-based education for melanoma recognition: A pilot study. Patient Educ Couns. 2018 Apr;101(4):738-742. | Participants not at high risk of melanoma |
| Martin RA, Weinstock MA, Risica PM, et al. Factors associated with thorough skin self-examination for the early detection of melanoma. J Eur Acad Dermatol Venereol. 2007 Sep;21(8):1074-81. | Participants not at high risk of melanoma |
| O'Connor C, Gallagher C, O'Connell M, et al. Bare necessities? The utility of full skin examination in the COVID-19 era. Clin Exp Dermatol. 2021 Jun;46(4):720-722. | Participants not at high risk of melanoma |
| Robinson JK, Stapleton J, Turrisi R. Relationship and partner moderator variables increase self-efficacy of performing skin self-examination. J Am Acad Dermatol. 2008 May;58(5):755-62. | Outcome not related to the early detection of melanoma |
| Robinson JK, Turrisi R, Stapleton J. Examination of mediating variables in a partner assistance intervention designed to increase performance of skin self-examination. J Am Acad Dermatol. 2007 Mar;56(3):391-7. | Outcome not related to the early detection of melanoma |
| Robinson JK, Wahood S, Ly S, et al. Melanoma detection by skin self-examination targeting at-risk women: A randomized controlled trial with telemedicine support for concerning moles. Prev Med Rep. 2021 Aug 26;24:101532. | Population setting |
| Sharma A, Ilyas M, Maganty N, et al. An effective game-based learning intervention for improving melanoma recognition. J Am Acad Dermatol. 2018 Sep;79(3):587-588. | Population setting |
| Smit AK, Espinoza D, Newson AJ, et al. A Pilot Randomized Controlled Trial of the Feasibility, Acceptability, and Impact of Giving Information on Personalized Genomic Risk of Melanoma to the Public. Cancer Epidemiol Biomarkers Prev. 2017 Feb;26(2):212-221. | Participants not at high risk of melanoma |
| Tsai S, Frank SH, Bordeaux JS. Improving Sun-Protective Behaviors and Self-Skin Examinations Among African Americans: A Randomized Controlled Trial. Dermatol Surg. 2018 Apr;44(4):512-518. | Participants not at high risk of melanoma |
| Walton AE, Janda M, Youl PH, et al. Uptake of skin self-examination and clinical examination behavior by outdoor workers. Arch Environ Occup Health. 2014;69(4):214-22. | Population setting |

^1^An additional 29 reports were excluded as they were not full reports of completed RCTs (including trial registrations, conference abstracts and protocols)

**Supplementary Table 3: Characteristics of included studies.**

| Study  Country | Population description | Intervention description | Control description | Primary outcome | Secondary outcomes |
| --- | --- | --- | --- | --- | --- |
| Ackermann 2022  Australia  [25] | Melanoma patients:  Stage 0 to II (n = 100) | Patient-led surveillance: usual follow up care with clinician plus reminders to perform SSE, patient-performed dermoscopy, teledermatologist assessment, and fast-tracked unscheduled clinic visits. (n = 49) | Clinician-led surveillance: usual follow up with clinician (n = 51) | Proportion of eligible and contacted patients who were randomised.  (6 months) | PROMs: SSE knowledge, attitudes and practices, psychological outcomes.  Clinical outcomes: clinic visits, skin surgeries, subsequent new primary or recurrent melanoma (6 months) |
| Bowen 2015  USA  [17] | Melanoma patients  (n = 311) | Web-based information and support: personal risk graphic, prevention information, SSE information, how to access health care and talk about risk, chat room, interactive section | No intervention (access to intervention at trial completion) | PROMs: SSE thoroughness including specific body areas examined. (12 months) | PROMs: Sun protection behaviour, Health provider screening techniques (12 months) |
| Bowen 2019  USA  [18] | First degree relatives of melanoma patients (n = 313) | Web-based information and support.  (n = 157) | No intervention (delayed access to intervention) (n = 156) | PROMs: SSE thoroughness including specific body areas examined. (12 months) | PROMs: Sun protection behaviours, health provider screening techniques.  (12 months) |
| Geller  2006  USA  [19] | Siblings of melanoma patients (n = 494) | Telephone counselling and personalised mailed materials: one goal setting call, three tailored printed mailings, three counselling calls. (n = 237) | Recommendation that melanoma patients notify family, encourage screening. (n = 257) | PROMs: Frequency and thoroughness of SSE.  (12 months) | PROMs: skin cancer screening examination by dermatologist, sun protection behaviours.  (12 months) |
| Geller  2023  USA  [24] | Childhood cancer survivors treated with radiotherapy.  (n=728) | Patient activation + Provider activation (n=245)  Patient activation + Provider activation + Teledermoscopy (n= 242) | Patient Activation: Educational materials, text messages, and access to a website. (n=241) | PROMs: Completion of a physician skin examination within 12 months post-intervention.  Conduct of SSE within 2 months prior to the 18-month assessment. | PROMs: mean number of body parts examined during self-examinations, assessed at baseline, 12, and 18 months |
| Glanz  2010  USA  [38] | At-risk primary care outpatients identified with BRAT (n = 724) | Personalised mailed skin cancer prevention and detection information. (n = 362) | Generic mailings. (n = 257) | PROMs: performance of SSE since baseline.  (4 months) | PROMs: Frequency of sun protection behaviours, Participant reaction to treatment and control materials (4 months) |
| Glanz  2015  USA  [39] | At-risk primary care patients identified with BRAT (n = 206) | Personalised mailed skin cancer risk prevention and detection information. (Updated materials) (n = 95) | Generic mailings (n = 111) | PROMs: performance of SSE since baseline.  (3 months) | PROMs: Sun protection behaviours, skin cancer screening exam by health care provider.  (3 months) |
| Manahan 2015  Australia  [35] | High risk melanoma patients (fair skin type, previous skin excisions, personal or family history).  (n = 58) | SSE plus mobile teledermoscopy plus detailed technical and SSE instructions (n = 29) | SSE plus mobile teledermoscopy plus detailed technical instructions only  (n = 29) | Sensitivity of SSE plus mobile teledermoscopy vs. in-person CSE using either patients or lesions as denominator; and concordance of telediagnosis with CSE. | Body sites examined, lesions photographed, and missed. |
| Manne 2010  USA  [20] | First degree relatives of melanoma patients (n = 443) | Personalised printed materials and telephone counselling: three print mailings tailored to participant risk and knowledge and one telephone counselling call. (n = 225) | Generic print and counselling: three print mailings, one telephone counselling call.  (n = 218) | PROMs: Skin cancer screening examination by doctor  (12 months) | PROMs: SSE frequency, sun protection habits  (12 months) |
| Manne 2021  USA  [27] | Melanoma patients: Stage 0 - III (n = 441) | mySmartSkin (MSS) web-based intervention targeting sun protection behaviours and skin self-examination.  (n = 224) | Usual care  (access to the MSS intervention at trial completion) (n = 217) | PROMs: Performance of thorough SSE defined as examining each body area during the most recent skin self-check. (6 months) | PROMs: Sun protection behaviours, Intervention mediators (knowledge, benefits, barriers, and self-efficacy for skin self-examination)  (6 months) |
| Manne 2022  USA  [34] | Patients at high risk of melanoma (personal/family history, high-risk phenotype).  (n = 116) | mySkinCheck automated web-based intervention targeting skin self-examination.  (n = 56) | Usual care:  (n = 60) | PROMs: SSE of 15 defined body parts in the last 3 months. | PROMs: SSE of any part of the body in the last 3 months and number of body parts examined during the last SSE. |
| Marce 2022  France  [21] | First degree relatives of melanoma patients  (n=280) | Tip sheet and oral advice given to melanoma patients for their first-degree relatives.  (n=166) | Oral advice only  n=114 | PROM: TCE with a dermatologist or a general practitioner performed within one year after advice | PROMs: SSE frequency, the planning of TCE (with or without a scheduled appointment), and the adoption of sun-protection behaviours by the FDR |
| Marek  2018  USA  [36] | Dermatology pigmented lesion clinic patients (n = 69) | Mobile app plus,  (1) the receipt of monthly skin exam reminders (n = 17), or (2) an accountability partner that would receive a monthly performance report of their skin exam progress (n = 17), or  (3) a combination of both monthly reminders and a report to an accountability partner (n = 17). | Mobile app only (n = 18) | PROMs: Change in SSE rates as assessed by enrolment and 6 month surveys.  (6 months) | PROMs: Patient satisfaction as assessed by the end-of-study survey.  (6 months) |
| Moncrieff 2022  Netherlands  UK  [16] | Melanoma patients: stage 1B-2C  (n = 388) | Stage adjusted reduced follow up frequency.  (n = 192) | Conventional follow up schedule as per national guidelines  (n = 196) | PROMs: Participant quality of life: STAI-S, CWS, IES, and RAND-36  (5 years) | Melanoma recurrence rates, site of recurrence, and method of detection. Disease-specific and overall survival  (5 years) |
| Murchie 2022  UK  [26] | Melanoma patients:  Stage 0-IIC  (n = 240) | Achieving Self-directed Integrated Cancer Aftercare (ASICA): a tablet based digital intervention to prompt and support skin self-examination in melanoma patients. Usual structured melanoma follow-up as determined by local guidelines.  (n = 121) | Usual structured melanoma follow-up as determined by local guidelines (n = 119) | Co-primary outcome measures:  Patient reported outcomes: Melanoma Worry Scale, anxiety and depression (HADS) and quality of life (EQ-5D-5L)  (12 months) | Patient reported outcomes: Adherence to recommendations for, self-efficacy in, and future intention and planning to perform SSE. Clinical outcomes: New primary and recurrent melanoma diagnoses.  Skin-related NHS resource use.  (12 months) |
| Oliveria 2004  USA  [37] | Patients with 5 or more dysplastic nevi. (n = 100) | Educational intervention and a photo book (personal whole-body photographs)  (n = 49) | Educational intervention only  (n = 51) | PROMs: Frequency and thoroughness of SSE (4 months) | PROMs: Skin cancer knowledge  (4 months) |
| Robinson 2007  USA  [28] | Melanoma patients  (n = 130) | Dyadic learning: Research assistant led demonstration of the ABCDE rule and skin self-examination skills training.  (n = 65) | Solo learning with the same intervention (n = 65) | Patient reported outcomes: skin self-examination performance and use of a body map to record areas of concern. (4 months) | Patient reported outcomes: Perceived self-efficacy in performing, perceived importance of, and intentions of performing skin self-examination.  (4 months) |
| Robinson 2010 USA  [29] | Melanoma patients:  Stage I or IIA (n = 40) | In person training involving partners. Enabling kit: ruler, magnifying lens, ABCDE rule card and body maps.  (n = 19) | Take home workbook plus enabling kit. (n = 21) | Patient reported outcomes: Frequency of partner assisted skin self-examination. (4 months) | Patient reported outcomes: self-efficacy in, attitudes to, and knowledge of skin self-examination  (4 months) |
| Robinson 2016  USA  [22] | Stage 0 to IIB melanoma patients and skin check partners. (n = 494) | Educational intervention delivered: (1) in-person (n = 165), or (2) take-home booklet intervention (n= 159), or (3) electronic interactive tablet (n = 71) | Usual care (n = 99) | Patient reported outcomes: Frequency of skin self-examination performance and easy- to-see and difficult-to-see regions (24 months) | Clinical: Detection of a new or recurrent melanoma by the dyad or physician, Number of unscheduled physician appointments for concerning lesions. (24 months) |
| Robinson 2020  USA  [23] | Melanoma patients and their partners (n = 341) | Phase 1: SSE Workbook in office, 4 monthly follow up with trial dermatologist, enabling kit (n = 134) Phase 2: Mailed SSE workbook, booklet of body maps and scorecards, enabling kit, community physician surveillance. (n= 63) | No intervention  (access to materials after trial completion) Phase 1 (n = 38) Phase 2 (n= 106) | Patient reported outcomes: skin self-examination frequency and body areas covered (18 months) | Patient reported outcomes: skin self-examination knowledge, importance, anxiety Clinical: Identification of concerning lesions determined by whether lesion was biopsied, detection of subsequent melanoma  (18 months) |
| Walter  2020  UK  [30] | GP patients identified as high risk by MelaTools risk assessment (n = 238) | Skin self-monitoring smartphone application with instructions for use and monthly reminders.  Nurse consultation with standard written advice on sun protection and skin cancer detection.  (n = 119) | Nurse consultation with standard written advice on sun protection and skin cancer detection. (n = 119) | Coprimary outcomes For any skin changes or pigmented lesions: (i) GP consultation rates and (ii) Patient interval (time between first noticing a change and consultation) | PROMs: SSE benefits and barriers; self-efficacy for consulting without delay; perceived melanoma risk; sun protection habits; MWS; HADS, quality of life, using the 12-item Short Form Health Survey scale. (12 months) |

If multiple reports were published from one trial, all reports contributed to data extraction, but the trial was included once.

PROMS: patient reported outcome measures

BRAT: brief skin cancer risk assessment tool

**Supplementary table 4: Recruitment and retention strategies by study**

| Study ID, Unit of allocation | Patient identification | Delivery of information | Recruitment strategies description | Retention or response strategies description. |
| --- | --- | --- | --- | --- |
| Ackermann 2022 | Nine physicians from 2 melanoma specialty clinics and 1 primary care skin cancer clinic identified potential participants during regular follow-ups. | In person or by mail | Pilot study testing feasibility of larger scale study – primary outcome is proportion of eligible and contacted patients who were randomised.  Invitation letter printed on clinic letterhead, signed by treating clinician.  Participants opt into the study by returning the signed consent form and participation card via reply-paid mail.  Can decline participation by returning the card, phone, or email.  If no response within two weeks, the research staff contacts participants via phone.  Non-responsive participants after the call are marked as “screen fails”.  Public and patient involvement representatives provided input on the study design and materials | Questionnaires:  Method of delivery: Mail or via an online link sent by email.  Follow up protocol: If the questionnaire isn't returned within a week, researchers sent up to five reminders by SMS or email, according to the participant's preferred contact method.  Additional Support: For persistent nonresponse, researchers offered telephone administration. |
| Bowen 2015  Bowen 2019  Family | 2 cancer databases | Mail followed by phone call. | Use of electronic health records to identify participants  Opt-out options after each of the steps of mailing information to the physicians, cases and FDR, prior to phone call to confirm eligibility.  Used diverse communication methods including telephone, email, voice mail, face to face contact, and written contact.  Modifications to enrolment and consent process to recruit families rather than individuals  Usability testing of final version, n=10  Evaluation of recruitment process published.  Bowen 2012 noted that despite vigorous efforts, unable to recruit lower income families as frequently. | Control group families received access to the study website at trial completion “delayed intervention” group  Explicitly determined participant had no treatment preference (intervention vs control) in screening phone call.  Immediate offer to complete baseline questionnaire in recruitment phone call. |
| Geller 2006  Family  (multiple siblings) | 4 dermatology clinics | Mail, follow up phone calls | Dermatologists were invited to recruit for the trial and had to meet criteria to participate.  Dermatologists approached index case and asked permission to contact siblings.  Siblings were mailed consent form and baseline questionnaire.  Non-respondents received a second mailing and a minimum of five follow-up calls. | Control group participants received written nontailored study materials at trial completion. Baseline questionnaires mailed, with repeat mailing and 5 follow up phone calls.  All participants received an incentive of two movie tickets or gift cards upon completion of each survey. |
| Geller 2023  Individual | Childhood cancer study participants (recruited from 27 clinical sites) | Mail | Use of electronic health records to identify participants.  Recruitment coincided with the release of the CCSS follow-up survey.  Consent was obtained verbally via the telephone, online, or by receipt of completed paper surveys. | Text message reminder for 18 month survey  Participants' self-reports were verified through chart reviews using a standard form sent to their providers.  Physician offices received $25.00 for this verification.  Sample size calculations included 25% attrition rate |
| Glanz 2010  Individual | 2 general practices | Face to face | Patients recruited in the waiting room, given brief screening questionnaire. | Participants were given small incentive gifts for returning surveys and diaries, such as magnets, pens, movie coupons, and movie coupons.  Exclusion criteria included: Self-reported plans not to be out of town for more than 2 weeks during the summer. |
| Glanz 2015  Individual | General practice | Face to face | Recruitment (enrolment and informed consent) by study staff in waiting room | Telephone interviews were conducted if respondents did not return the mailed survey after two reminders.  Incentives $20 for each survey  Exclusion criteria: Planning to be out of town for more than three consecutive weeks during the next three months |
| Manahan 2015  Individual | Cohort study (QSkin), volunteers | Mail | Incentive: Participants were reimbursed with a $100(AU) voucher to cover costs and time.  Participants completed a questionnaire on teledermatology acceptance, whether they had access to an iPhone, and whether they would be interested in participating in a study of SSE plus mobile teledermoscopy  Screened 559 potential participants but only needed a sample size of 50 | Sample size included attrition rate of 15% |
| Manne 2010  Family | 3 cancer clinic registries | Mail followed by phone call. | Use of electronic health records to identify participants  Consent process adapted for FDR | Participants were given a $10 gift certificate for each assessment completed.  Physician confirmation of total skin examination - data verification. |
| Manne 2021  Individual | Dermatology/cancer clinics and cancer database. | Mail followed by phone call. | Recruitment method was varied according to site.  Potentially eligible patients were mailed a study information letter and consent form.  Research team followed up via telephone to gauge interest and eligibility.  Second mailing sent if necessary.  Treating physicians were contacted to ensure suitability before approaching patients. Pre-trial planning - detailed sample size calculations considering both primary and secondary outcomes  User testing, n=15 | Control group participants offered access to the mySmartSkin intervention at end of study Participants sent birthday and non-denominational holiday cards to maintain their interest and engagement in the study.  Online surveys  $25 per completed survey  10% attrition estimated and incorporated into sample size calculations. |
| Manne 2022  Individual | High risk melanoma clinic or self-referral from clinical trials.gov | Face to face in clinic or mailed for self-referrals. | During a clinic visit, patients were informed of the study by the dermatologist.  Patients were given a brochure, consent form and contacts for the research team.  Participants were also able to self-refer after learning about the study at www.clinicaltrials.gov and were sent the flyer and consent form by email or postal mail. | Online baseline and follow up surveys.  $20 gift card for completing each survey.  Participants were prompted via automated email, telephone, text message and mail, as necessary, to complete the online surveys. |
| Marce 2022  Family | 9 hospital centres | Index cases – face to face  FDR: telephone, email or phone | Cluster RCT: Heads of dermatology departments gave their consent for their sites to participate in the study.  Melanoma patients were identified at initial diagnosis.  Intervention Group: Received oral advice and a tip sheet for their FDRs.  Control Group: Received only oral advice.  At 1 year follow up visits, index cases were asked to participate and provide FDR contact details.  FDRs were contacted by telephone, email, or mail if not reachable by phone.  FDRs expressing non-opposition were included in the study. | Primary and secondary outcomes were collected via phone interviews by a clinical research assistant.  Assistant received training to perform data collection.  Calls were centralised to standardise outcome assessment.  To verify the reliability of self-reported TCE, a random sample of 50 FDRs (25 from each trial group) was selected, and their physicians were contacted to confirm the reported TCE consultations |
| Marek 2018  Individual | Pigmented lesion clinic | Face to face | Pre-trial planning - targeted enrolment of 70 patients based on prior literature successfully assessing the impact of image-based interventions on SSE rates  Face to face enrolment of patients presenting for visit. | Nil reported |
| Moncrieff 2022  Individual | Dermatology outpatients in Netherlands and UK | Face to face | Face to face recruitment at clinic follow up visit  Physician or nurse practitioner delivered information about the trial face to face. | Exclusion criteria: those unable to complete the questionnaires.  Mailed questionnaires - provide pre-stamped return envelope. |
| Murchie 2022 Individual | Hospital clinic and pathology registers, meeting lists | Mail followed by choice of phone call or face to face visit. | Nonrandomised feasibility study (n=19) provided information on recruitment. Eligible patients identified by the local clinical team using electronic and paper records  Invitation letter, patient information leaflet, baseline questionnaire, consent form, and SAE sent out to possible participants from the treating hospital.  Possible participants offered the opportunity to discuss their participation in the project face-to-face or on the telephone with a member of the ASICA team.  Baseline questionnaire and consent form returned by post.  Codesign of the intervention with potential recipients.  Non-randomised feasibility study (n=19) established acceptability | Reminders sent to non-responders after 3 weeks.  In the case of non-return of questionnaires, attempts made by staff to trace the participant directly using preferred method for prompts or indirectly by contacting the GP.  Collection of routine data from health records  Eligibility criteria includes inability to complete questionnaires |
| Oliveria 2004  Individual | Dermatology clinic | Face to face | Recruited and informed consent obtained at scheduled clinic visit. Recruited by physician or nurse. Face to face | Surveys completed at face to face visits |
| Robinson 2007  Individual | Hospital database | Not reported | Participants were drawn from a hospital registry of 682 patients who were given a diagnosis of CM and were being seen at least annually by a physician for their skin conditions.  Potential study participants were contacted directly by a research assistant at the time of a physician visit.  "nominal payment" | Participants in both the control and treatment groups were administered a SSE assessment questionnaire before the intervention, immediately after the intervention, and at a 4-month follow-up  visit. |
| Robinson 2010  Individual | Not reported | Not reported | "Subjects were offered nominal payment" | Nil |
| Robinson 2016  Dyad | Hospital database, volunteers | Mail followed by phone call. | Patients identified by electronic medical records of the Northwestern Medicine health care system.  Advertisements were placed for 12 weeks in the health sections of 2 regional newspapers with large circulations Both melanoma survivors and their skin check partners provided written informed consent  The histopathologic report was reviewed by the research assistant and the dermatologist to ensure that the patient had a melanoma that met inclusion criteria.  Tablet usability survey given to the first 15 pairs enrolled Sample size based on power calculations and estimated 20% attrition rate. (B6) | Patients and partners received $20 to complete each assessment,  Questionnaires completed at face to face assessment visit. Exclusion criteria: unable to commit to skin exams by study dermatologist every 4 months for 24 months.  Estimated 20% attrition when calculating sample size |
| Robinson 2020  Dyad | Hospital repository of patients willing to participate in research, Robinson 2016 trial participants. | Mixed: face to face, letter/telephone call | Participants identified through the Enterprise Data Warehouse (EDW) of Northwestern University, a repository of patients willing to participate in research. Participants also identified from Robinson 2016.  Multiple methods of recruitment: face to face and letter/phone call Both melanoma survivors and their skin check partners provided written informed consent.  Pathology confirmed prior to enrolment and included in eligibility criteria Sample size rationale and estimation included. | Exclusion criteria includes being unable to commit to complete online surveys at 9 months and 18 months.  Estimated 20% attrition in sample size calculation At the end of the Phase 2 study, pairs randomized to the control condition received the intervention materials.  Both melanoma survivors and skin check partners received $20 in Phase 1 and $25 in Phase 2 to complete each survey. Phase 1, pairs completed the self-report survey at an in-person visit every 4 months.  Phase 2, data collection online |
| Walter 2020  Individual | 12 general practices | Face to face | Researchers recruited participants opportunistically in the reception area.  Protocol specifies recruitment at different times of the day and different days of the week, in order to approach people of different ages, gender and educational level. Participants were reimbursed with a voucher for £10. General practices reimbursed to cover administrative, or time costs associated with supporting the study.  Public and patient involvement representatives provided input on the study design and materials.  Earlier qualitative research provided understanding of consumer views. | Baseline questionnaire completed in person Follow up questionnaires - email link to online questionnaire.  Up to 2 reminders sent (email) for 6 month and 12 month questionnaire  Audit of GP records - routine data used |

**Supplementary figure 1: MEDLINE Search Strategy**

1 exp Melanoma/ (105046)

2 exp Skin Neoplasms/ (138826)

3 melanoma*.tw. (129922)

4 1 or 2 or 3 (240828)

5 exp Self Care/ (60287)

6 exp Self-Management/ (4634)

7 exp Self-Examination/ (2739)

8 exp "Early Detection of Cancer"/ (34246)

9 exp Telemedicine/ (40972)

10 Patient Education as Topic/ (88082)

11 telederm*.tw. (1199)

12 (digital adj2 (dermoscop* or dermatoscop*)).tw. (329)

13 ((mobile or cell or cellular or smart) adj ((phone* adj2 app*) or application*)).tw. (6770)

14 smartphone.tw. (15014)

15 total body photography.tw. (105)

16 self-examination.tw. (3127)

17 (skin adj3 examination*).tw. (4883)

18 SSE.tw. (1547)

19 (self adj3 exam*).tw. (11515)

20 self-monitor*.tw. (9254)

21 self-manag*.tw. (24507)

22 patient-led.tw. (634)

23 surveillance.tw. (212052)

24 5 or 6 or 7 or 8 or 9 or 10 or 11 or 12 or 13 or 14 or 15 or 16 or 17 or 18 or 19 or 20 or 21 or 22 or 23 (473721)

25 randomized controlled trial.pt. (572058)

26 controlled clinical trial.pt. (94928)

27 randomized.ab. (567020)

28 placebo.ab. (229628)

29 clinical trials as topic.sh. (200137)

30 randomly.ab. (385891)

31 trial.ti. (265580)

32 25 or 26 or 27 or 28 or 29 or 30 or 31 (1457225)

33 exp animals/ not humans.sh. (5023830)

34 32 not 33 (1340356)

35 4 and 24 and 34 (375)

**Supplementary figure 2: Full text screening flow chart**

| **Lead author name and Ref ID Number:** | | | |
| --- | --- | --- | --- |
| **Design:**  Eligible: RCTs  Ineligible: Reviews (although these should be recorded and used as a source of references), non-randomised trials, observational studies, conference abstracts, trial registrations, protocols of RCTs (without report of actual RCT) | Yes  ↓  next question | Unclear  ↓  next question | No  →  EXCLUDE  reason:  Not a report of a completed RCT |
| **Setting:**  Eligible: clinical care, clinic and hospital databases.  Ineligible: population setting. | Yes  ↓  next question | Unclear  ↓  next question | No  →  EXCLUDE  reason:  Population setting |
| **Population:**  Eligible: personal history of melanoma, family history of melanoma, atypical/dysplastic naevi, personal risk factors may include high naevi count, phenotypic traits, high sun exposure.  Ineligible: healthy volunteers, general population, participants undergoing current treatment for melanoma | Yes  ↓  next question | Unclear  ↓  next question | No  →  EXCLUDE  reason:  Participants not at high risk of melanoma |
| **Intervention:**  Eligible: intervention related to the early detection of melanoma  Ineligible: drug intervention | Yes  ↓  next question | Unclear  ↓  next question | No  →  EXCLUDE  reason:  Intervention not related to early detection of melanoma |
| **Outcomes**:  Eligible: the early detection of melanoma or surrogate for this (e.g., improved skin self-examination practice)  Ineligible: Outcomes relate only to primary prevention such as change in sun protection behaviours. | Yes  ↓  next question | Unclear  ↓  next question | No  →  EXCLUDE  reason:  Outcome not related to the early detection of melanoma or a surrogate for this. |
| **Final Decision** | **INCLUDE** | **UNCLEAR**  **(Discuss)** | **EXCLUDE** |

**Supplementary Figure 3: Preferred Reporting Items for Systematic Reviews and Meta-Analyses Flow diagram**

**Identification of studies via databases**

Records removed *before screening*:

- Duplicate records removed by Covidence automation tool.

(n = 700)

Records identified from:

Databases (n = 1746)

- Central (n = 384)
- CINAHL (n = 146)
- Embase (n = 794)
- Medline (n = 421)

**Identification**

Records screened

(n = 1046)

Records excluded by reviewers

(n = 964)

Reports sought for retrieval

(n = 82)

Reports excluded: (n = 54)

- Not a report of a completed RCT (n = 29)
- Population setting (n = 10)
- Participants not at high risk of melanoma (n = 8)
- Outcome not related to the early detection of melanoma or a surrogate for this (n = 5)
- Intervention not related to early detection of melanoma (n = 2)

**S**

**Screening**

Reports assessed for eligibility

(n = 82)

Studies included in review.

(n = 21)

(reported across 28 papers)

**Included**

*From:*  Page MJ, McKenzie JE, Bossuyt PM, Boutron I, Hoffmann TC, Mulrow CD, et al. The PRISMA 2020 statement: an updated guideline for reporting systematic reviews. BMJ 2021;372:n71. doi: 10.1136/bmj.n71. For more information, visit: <http://www.prisma-statement.org/>

**Preferred Reporting Items for Systematic reviews and Meta-Analyses extension for Scoping Reviews (PRISMA-ScR) Checklist**

| **SECTION** | **ITEM** | **PRISMA-ScR CHECKLIST ITEM** | **REPORTED**  **ON PAGE #** |
| --- | --- | --- | --- |
| **TITLE** | | | |
| Title | 1 | Identify the report as a scoping review. | 1 |
| **ABSTRACT** | | | |
| Structured summary | 2 | Provide a structured summary that includes (as applicable): background, objectives, eligibility criteria, sources of evidence, charting methods, results, and  conclusions that relate to the review questions and objectives. | 3 |
| **INTRODUCTION** | | | |
| Rationale | 3 | Describe the rationale for the review in the context of what is already known. Explain why the review  questions/objectives lend themselves to a scoping review approach. | 4 |
| Objectives | 4 | Provide an explicit statement of the questions and objectives being addressed with reference to their key elements (e.g., population or participants, concepts, and context) or other relevant key elements used to  conceptualize the review questions and/or objectives. | Supplementary table 1 |
| **METHODS** | | | |
| Protocol and registration | 5 | Indicate whether a review protocol exists; state if and where it can be accessed (e.g., a Web address); and if available, provide registration information, including the registration number. | 5 |
| Eligibility criteria | 6 | Specify characteristics of the sources of evidence used as eligibility criteria (e.g., years considered, language,  and publication status), and provide a rationale. | Supplementary table 1, 6 |
| Information sources* | 7 | Describe all information sources in the search (e.g., databases with dates of coverage and contact with authors to identify additional sources), as well as the date the most recent search was executed. | 5 |
| Search | 8 | Present the full electronic search strategy for at least 1  database, including any limits used, such that it could be repeated. | Supplementary figure 3: PRISMA diagram |
| Selection of sources of evidence† | 9 | State the process for selecting sources of evidence (i.e., screening and eligibility) included in the scoping review. | 5-6  Supplementary figure 2: Full text screening flow chart |
| Data charting process‡ | 10 | Describe the methods of charting data from the included sources of evidence (e.g., calibrated forms or forms that have been tested by the team before their use, and whether data charting was done independently or in duplicate) and any processes for obtaining and  confirming data from investigators. | 6 |
| Data items | 11 | List and define all variables for which data were sought and any assumptions and simplifications made. | 6 |
| Critical appraisal of individual sources of evidence§ | 12 | If done, provide a rationale for conducting a critical appraisal of included sources of evidence; describe the  methods used and how this information was used in any data synthesis (if appropriate). | N/A |
| Synthesis of results | 13 | Describe the methods of handling and summarizing the data that were charted. | 6 |

| **SECTION** | **ITEM** | **PRISMA-ScR CHECKLIST ITEM** | **REPORTED**  **ON PAGE #** |
| --- | --- | --- | --- |
| **RESULTS** | | | |
| Selection of sources of evidence | 14 | Give numbers of sources of evidence screened, assessed for eligibility, and included in the review, with  reasons for exclusions at each stage, ideally using a flow diagram. | 6, Supplementary figure 3: PRISMA diagram |
| Characteristics of sources of evidence | 15 | For each source of evidence, present characteristics for which data were charted and provide the citations. | 6-7  Supplementary table 3: Characteristics of included studies |
| Critical appraisal within sources of evidence | 16 | If done, present data on critical appraisal of included sources of evidence (see item 12). | N/A |
| Results of  individual sources of evidence | 17 | For each included source of evidence, present the  relevant data that were charted that relate to the review questions and objectives. | Supplementary table 4: Recruitment and retention strategies by study |
| Synthesis of results | 18 | Summarize and/or present the charting results as they relate to the review questions and objectives. | 7-14, Figures 1, 2, Tables 1-4 |
| **DISCUSSION** | | | |
| Summary of evidence | 19 | Summarize the main results (including an overview of concepts, themes, and types of evidence available), link to the review questions and objectives, and consider the relevance to key groups. | 14 |
| Limitations | 20 | Discuss the limitations of the scoping review process. | 17-18 |
| Conclusions | 21 | Provide a general interpretation of the results with respect to the review questions and objectives, as well  as potential implications and/or next steps. | 17-18 |
| **FUNDING** | | | |
| Funding | 22 | Describe sources of funding for the included sources of evidence, as well as sources of funding for the scoping  review. Describe the role of the funders of the scoping review. | 1 |

JBI = Joanna Briggs Institute; PRISMA-ScR = Preferred Reporting Items for Systematic reviews and Meta-Analyses extension for Scoping Reviews.

* Where *sources of evidence* (see second footnote) are compiled from, such as bibliographic databases, social media platforms, and Web sites.

† A more inclusive/heterogeneous term used to account for the different types of evidence or data sources (e.g., quantitative and/or qualitative research, expert opinion, and policy documents) that may be eligible in a scoping review as opposed to only studies. This is not to be confused with *information sources* (see first footnote).

‡ The frameworks by Arksey and O’Malley (6) and Levac and colleagues (7) and the JBI guidance (4, 5) refer to the process of data extraction in a scoping review as data charting*.*

§ The process of systematically examining research evidence to assess its validity, results, and relevance before using it to inform a decision. This term is used for items 12 and 19 instead of "risk of bias" (which is more applicable to systematic reviews of interventions) to include and acknowledge the various sources of evidence that may be used in a scoping review (e.g., quantitative and/or qualitative research, expert opinion, and policy document).

*From:* Tricco AC, Lillie E, Zarin W, O'Brien KK, Colquhoun H, Levac D, et al. PRISMA Extension for Scoping Reviews (PRISMAScR): Checklist and Explanation. Ann Intern Med. 2018;169:467–473. [doi: 10.7326/M18-0850.](http://annals.org/aim/fullarticle/2700389/prisma-extension-scoping-reviews-prisma-scr-checklist-explanation)
